# Placental Transfer of Maternally-Derived IgA Precludes the Use of Guthrie Card Eluates as a Screening Tool for Primary Immunodeficiency Diseases

**DOI:** 10.1371/journal.pone.0043419

**Published:** 2012-08-16

**Authors:** Stephan Borte, Magdalena Janzi, Qiang Pan-Hammarström, Ulrika von Döbeln, Lennart Nordvall, Jacek Winiarski, Anders Fasth, Lennart Hammarström

**Affiliations:** 1 Division of Clinical Immunology and Transfusion Medicine, Department of Laboratory Medicine, Karolinska Institutet at Karolinska University Hospital Huddinge, Stockholm, Sweden; 2 Translational Centre for Regenerative Medicine (TRM), University of Leipzig, Leipzig, Germany; 3 ImmunoDeficiencyCenter Leipzig (IDCL) at Hospital St. Georg gGmbH Leipzig, Jeffrey Modell Diagnostic and Research Center for Primary Immunodeficiencies Leipzig, Leipzig, Germany; 4 Division of Metabolic Diseases, Department of Laboratory Medicine, Karolinska Institutet at Karolinska University Hospital Huddinge, Stockholm, Sweden; 5 Department of Women’s and Children’s Health, Uppsala University, Academic Hospital, Uppsala, Sweden; 6 Division of Pediatrics (CLINTEC), Karolinska Institutet at Karolinska University Hospital Huddinge, Stockholm, Sweden; 7 Department of Pediatrics, The Sahlgrenska Academy, University of Gothenburg, Gothenburg, Sweden; University of Cape Town, South Africa

## Abstract

There is a need for neonatal screening tools to improve the long-term clinical outcome of patients with primary immunodeficiency diseases (PID). Recently, a PCR-based screening method for both TRECs and KRECs using Guthrie card samples has been developed. However, the applicability of these excision circle assays is limited to patients with severe T or B cell lymphopenia (SCID, XLA and A-T), whereas the most common forms of PID are not detected. Absence of serum IgA is seen in a major fraction of patients with immunological defects. As serum IgA in newborns is considered to be of fetal origin, eluates from routinely collected dried blood spot samples might thus be suitable for identification of children with PID. To assess the applicability of such screening assays, stored Guthrie card samples were obtained from 47 patients with various forms of primary immunodeficiency diseases (SCID, XLA, A-T, HIGM and IgAD), 20 individuals with normal serum IgA levels born to IgA-deficient mothers and 51 matched healthy newborns. Surprisingly, normal serum IgA levels were found in all SCID, XLA, A-T and HIGM patients and, additionally, in all those IgAD patients born to IgA-sufficient mothers. Conversely, no serum IgA was found in any of the 16 IgAD patients born by IgA-deficient mothers. Moreover, half of the IgA-sufficient individuals born by IgA-deficient mothers also lacked IgA at birth whereas no IgA-deficient individuals were found among the controls. IgA in neonatal dried blood samples thus appears to be of both maternal and fetal origin and precludes its use as a reliable marker for neonatal screening of primary immunodeficiency diseases.

## Introduction

During pregnancy, the fetus depends on maternal transfer of specific antibodies for protection against pathogens. Humans produce five major immunoglobulin classes (IgG, IgA, IgM, IgE, IgD) and IgG is the only isotype that is actively transported from mother to child [1]–[9]. Several studies have previously demonstrated the presence of IgA in cord blood [1], [10]–[15] and IgA-positive B cells have also been reported in fetal tissues [16], [17] as well as in cord blood [18]–[21], suggesting that the IgA detected in neonatal blood is exclusively of fetal origin.

Primary immunodeficiency diseases (PID) comprise a group of more than 200 inherited genetic disorders caused by defects of innate and adaptive immune function [22]. The clinical severity ranges from non-symptomatic to recurrent, and potentially fatal, infections. Major efforts are currently undertaken to develop methods for neonatal PID screening, as early diagnosis and treatment would prevent subsequent tissue damage and premature death.

Defects in humoral immunity account for more than 60% of all forms of PID. The most common disorder, selective IgA deficiency (IgAD), is defined as serum IgA levels at or below 0.07 g/L with normal IgM and IgG levels in individuals of four years of age or older [23]. The estimated prevalence of IgAD is one in 600 in Caucasians [24]. Low or absent serum IgA is also included in the phenotype of a majority of other forms of PID (Table 1). Thus, lack of serum IgA at birth could potentially serve as a condition that would allow neonatal screening of various forms of PID.

**Table 1:** IgA levels and total T cell count for a selection of PID with IgA deficiency included in the phenotype.

Disease		Estimated incidence	T lymphopenia present at birth‡	IgA deficiency observed in the clinical phenotype†
IgAD		1∶600* [24]	No [23]	Yes [23]
CVID		1∶20–50.000* [29]	No [23]	Yes [30]
HIGM		1∶300.000 [31]	No [23]	Yes [23]
XLA		1∶70–100.000 [32]	No [22], [23]	68 in 103 reported cases (66%) [33]–[45]
A-T		1∶100.000 [46]	Yes [26], [27]	243 in 420 reported cases (58%) [47]–[79]
SCID	*IL2RG* (X-SCID)	1∶200.000 [46]	Yes [26]	11 in 12 reported cases (92%) [80]–[89]
SCID	*JAK3*	1∶500.000 [46]	Yes [26]	12 in 15 reported cases (80%) [90]–[94]
SCID	*IL7R*	not known [46]	Yes [26]	15 in 24 reported cases (62%) [91], [95]–[100]
SCID	*RAG1*	1∶100.000 [46]	Yes [26]	20 in 45 reported cases (44%) [89], [101]–[109]
SCID	*RAG2*	1∶100.000 [46]	Yes [110]	15 in 20 reported cases (75%) [102], [111]
SCID	*ADA*	1∶200–1.000.000 [46]	Yes [110]	23 in 40 reported cases (57%) [112]–[123]
DiGeorge syndrome with relevantfeatures of immunodeficiency‡ †		1∶15.000 [27]	Yes [27]	9 in 17 reported cases (53%) [124]–[133]

* Adapted from the prevalence seen in adults.

‡ Clinically relevant T cell lymphopenia defined as CD3^+^ T cell counts <500/mm^3^.

† Serum IgA levels <0.07 g/L.

In the 1960s, several countries introduced newborn screening programmes (NBS) for phenylketonuria, using eluates from dried blood spot samples (DBSS) of Guthrie cards. Other metabolic disorders have subsequently been added to the NBS programmes and today this screening constitutes an established form of preventive healthcare. In Sweden, DBSS have been used for NBS since 1965 and samples have been stored since 1975. As shown in our previous study [25], serum proteins can easily be eluted from stored DBSS and the corresponding levels be determined by sandwich ELISA or serum microarray techniques.

Although current neonatal PCR-based screening methods, using DNA extracted from Guthrie cards to quantify T-cell receptor excision circles (TRECs) and kappa-deleting recombination excision circles (KRECs), identify a majority of patients with severe combined immunodeficiencies (SCID) and X-linked agammaglobulinemia (XLA) [26], [27], patients suffering from the most prevalent forms of PID cannot be diagnosed using this method.

The aim of the present study was therefore to evaluate if lack of serum IgA in routinely collected DBSS eluates could serve as a condition to screen for PID.

## Results

### Elution Efficacy of IgA from Dried Blood Spot Samples

To determine the most efficient elution procedure for IgA from DBSS, serum IgA levels of freshly collected blood specimens were compared with the IgA levels of eluates from dried blood filter cards, prepared using the same blood samples. The serum levels of IgA were determined by routine nephelometry and ranged between 1.5–4.5 g/L, while the IgA levels in the DBSS eluates were determined by ELISA. On average, 70–85% of the serum IgA could be recovered from DBSS eluted at 4°C for 7 days (Table 2). Similar results were obtained for IgG levels in DBSS eluates (data not shown). For reasons of practicability and considering the potential risk of protein degradation at elevated temperatures, we chose to elute the DBSS at 4°C.

**Table 2:** Percentage agreement of the IgA elution from DBSS by ELISA compared to reference serum IgA levels measured by nephelometry.

Elutioncondition	4°Ctemperature	22°Ctemperature	37°Ctemperature
1 hour	78%	82%	61%
24 hours	87%	93%	90%
48 hours	84%	85%	86%
7 days	86%	86%	69%

### Long-time Storage does not Preclude Successful Elution of IgA from DBSS

**Figure 1:**
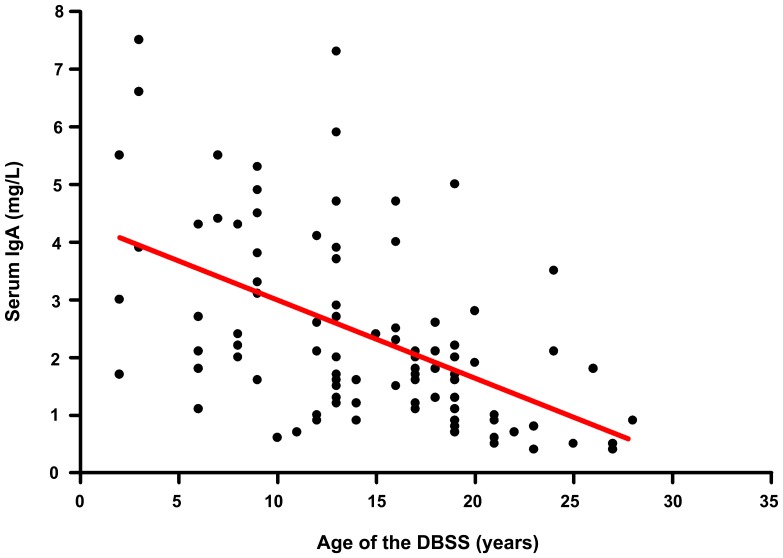
Impact of the storage time on the detection of IgA levels in DBSS eluates. The overall correlation is shown as a red trend line (ρ = −0.54). DBSS with undetectable IgA levels are not shown.

Ninety-two of the 118 DBSS included in the study had detectable IgA levels (51 controls, 31 PID patients and 10 IgA-sufficient children born to IgAD mothers) and could be analyzed to assess the effect of storage time prior to elution on the amount of eluted IgA (Figure 1). All DBSS were taken from original Guthrie cards routinely kept in sealed bags at 4°C–8°C for long-time storage. Correlation analysis of IgA levels and storage times indicates that the amount of eluted and/or detected IgA slightly decreases as a function of longer storage time (ρ = −0.54, p<0.0001). However, considerable IgA levels could be detected even in samples stored for 28 years, providing evidence that long-time storage does not exclude samples from analysis, nor would markedly influence the results obtained. Similar results were obtained previously for other serum proteins eluted from DBSS, that had been stored for long time [25].

### Reference Values for IgA Levels in DBSS Eluates from Healthy Newborns

**Figure 2:**
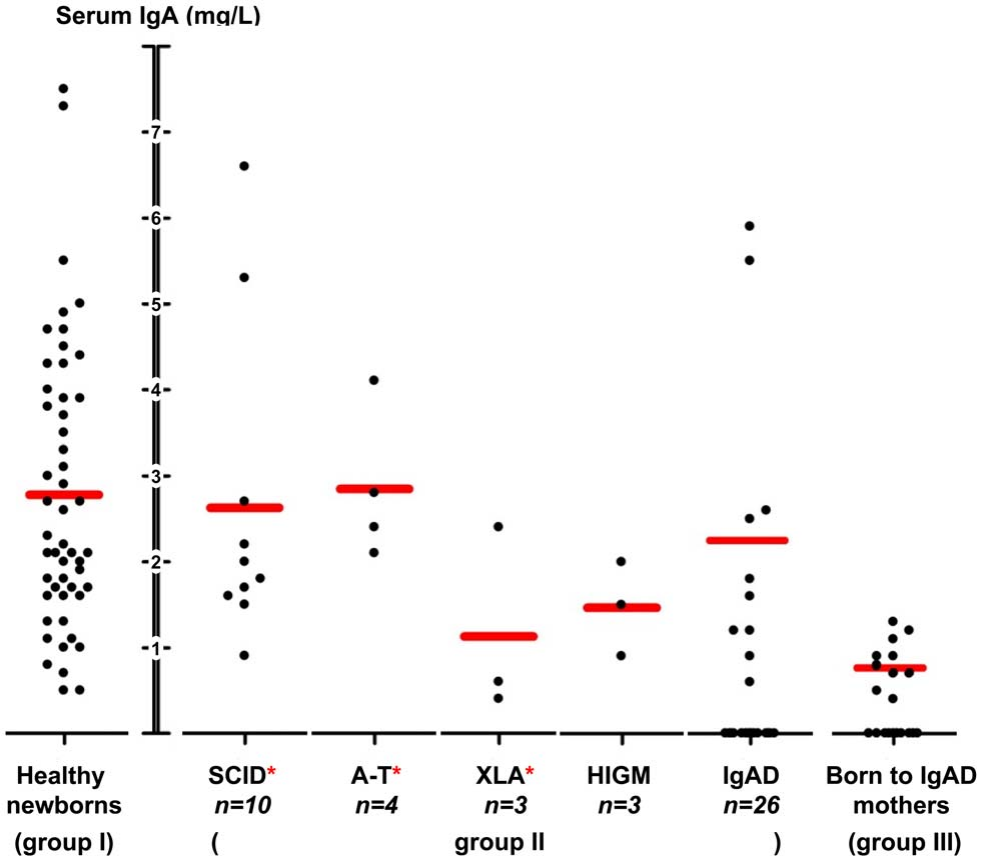
IgA levels in eluates from neonatal DBSS. Mean IgA levels per group are marked as horizontal red bars. One SCID patient was excluded due to extremely high levels of IgA in the DBSS eluate. SCID, A-T and XLA DBSS have previously shown to result in abnormal test results in the TREC/KREC assay and have been indicated by red asterisks [26], [27].

All DBSS eluates had detectable IgA levels in healthy newborns (group I), with a mean of 2.8 mg/L (range: 0.5–7.5 mg/L, Figure 2). These levels are comparable to those obtained from umbilical cord blood at delivery, as reported by Malek and colleagues [14]. For 8 individuals from this group, a separate elution from a second punch of the original Guthrie card was performed to re-assess the initially measured IgA levels, returning consistent results with a high degree of correlation (r^2^ = 0.97).

### Presence of IgA in Neonatal DBSS from Patients with SCID, XLA, HIGM or A-T

Within the group of PID patients (group II), all the patients diagnosed with SCID, XLA, Hyper IgM syndrome (HIGM) or Ataxia-telangiectasia (A-T) (n = 21) had detectable levels of serum IgA at birth, although all of them are IgA-deficient today (n = 10) or at the time of hematopoietic stem cell transplantation (n = 11). Moreover, all IgAD patients born to mothers with normal serum IgA levels also had detectable levels of IgA in neonatal DBSS (n = 10). The mean serum IgA level of these PID patients with detectable IgA levels at birth was 2.3 mg/L (range: 0.4–6.6 mg/L). A summary of the IgA levels based on specific disorders in group II is given in Figure 2, including previously reported classification results of these patients in the TREC/KREC assay [26], [27].

**Figure 3:**
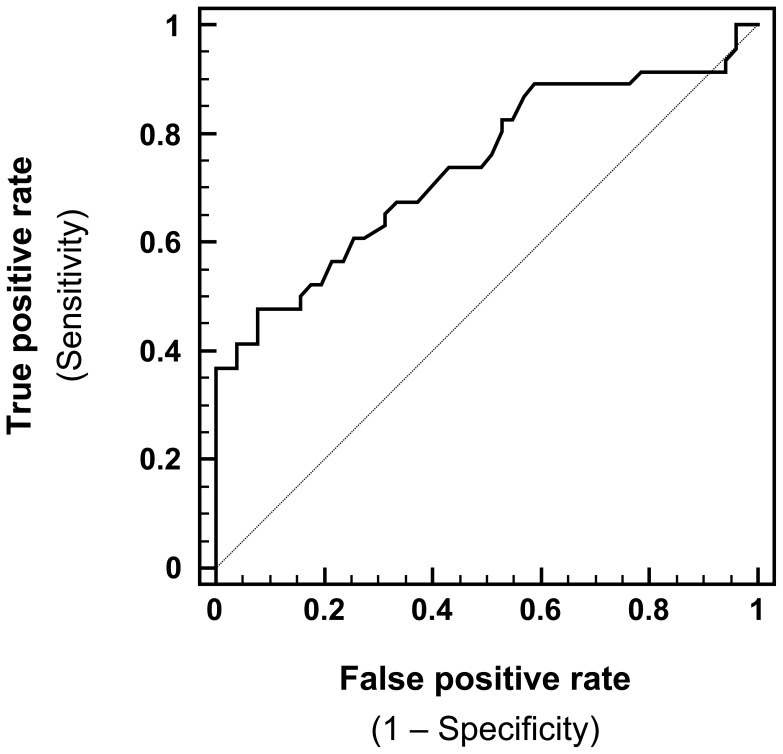
ROC curve plot depicting the performance of serum IgA levels in DBSS to predict IgA-deficient newborns (group II) compared with healthy newborns (group I).

The test characteristics for measuring IgA levels in neonatal DBSS to predict IgA-deficient PID patients are shown in Table 3 based on cutoff values that were chosen by receiver operating characteristic (ROC) curve analysis (Figure 3).

**Table 3:** Descriptive test characteristics of measuring IgA levels in DBSS to predict IgA-deficient newborns (group II).

Cutoff value*	Sensitivity	Specificity	PPV	NPV
≤0.35	0.39	1.0	100%	63%
≤0.6	0.41	0.96	90%	64%
≤0.9	0.47	0.92	84%	66%
≤1.2	0.5	0.84	74%	65%

* Serum IgA [mg/L]; PPV: positive predictive value; NPV: negative predictive value. PPV and NPV calculations are based on cumulative prevalence estimates from adults with diseases represented in group II.

### Maternal IgA-deficiency is Associated with Lack of IgA in DBSS Eluates

Sixteen of 26 samples (62%) from patients diagnosed with IgAD were found to have undetectable levels of IgA at birth (Figure 2), yet all featured normal IgG levels (9–18 g/L; data not shown), indicating that the samples were eluted properly. Interestingly, all these IgAD patients identified with undetectable IgA levels in DBSS eluates were born to IgA-deficient mothers, suggesting an impact of maternal IgAD on the IgA levels in the newborns. All of these IgAD patients were previously shown to have normal TREC and KREC copy numbers (Figure 2) [26], [27].

To test for a maternal influence on fetal IgA levels, DBSS from 20 children with normal IgA levels at follow-up (1 to 16 years of age), born to IgAD mothers, were collected and analyzed (group III). Ten of these children (50%) also showed undetectable serum IgA levels at birth.

In the remaining 10 samples from healthy children born to IgAD mothers (group III), IgA could be detected with a mean level of 0.9 mg/L (range: 0.4–1.3 mg/L, Figure 2). This constitutes about 33% of the mean level in healthy newborns (group I), suggesting that maternal IgA contributes at least 67% of the IgA found in DBSS eluates.

## Discussion

Novel neonatal screening methods for simultaneous detection of SCID and XLA patients, based on quantitation of TRECs and KRECs, have recently been proposed [26], [27]. However, these methods only identify a small proportion of all PID patients. As IgA-deficiency is included not only in the phenotype of most antibody deficiencies, albeit to varying degrees (Table 1), but also in a majority of other forms of PID, determination of serum IgA levels could potentially serve as a basis for screening programmes of inborn immune defects.

Although such a proposed screening for neonatal IgA-deficiency would result in the identification of patients with IgAD, these patients are not the primary target group for this screening as it represents a clinically mild form of PID. A screening test for IgAD would rather constitute a first step in the immunological evaluation, to be followed by second-tier tests and more in-depth analyses to establish the correct diagnosis.

The material for such a screening might preferably be a dried blood spot sample from regular Guthrie cards, as these are already routinely collected in a number of countries. In one of our previous studies [25], we investigated the possibility of identifying patients with primary immunodeficiency diseases of the complement system using DBSS. Complement C3 was readily detected in DBSS eluates from healthy controls using both sandwich ELISA and reverse phase serum microarrays. In contrast, DBSS eluates from patients with C3 deficiency lacked the protein. Thus, proteins eluted from Guthrie card samples may well be used in a high-throughput setting to identify individuals with selected primary immunodeficiency diseases.

As IgG is the only class of immunoglobulins that is actively being transported from mother to child during pregnancy [1]–[9], serum IgA in newborns is considered to be of fetal origin.

To investigate the possibility to diagnose PID at birth, we thus measured serum IgA levels in DBSS eluates from patients with various forms of PID. None of the patients with SCID, HIGM, XLA or A-T lacked IgA at birth and IgAD patients born to IgA-sufficient mothers all had detectable IgA levels at birth. In contrast, serum IgA was absent in the DBSS of IgAD children born to IgA-deficient mothers, indicating that IgA deficiency is present already at birth. In addition, an association between maternal and fetal levels of IgA was also seen in IgA-sufficient children born to IgA-deficient mothers, as 10 out of 20 children lacked IgA at birth.

Based on our results, we therefore propose the existence of a hitherto not fully appreciated mechanism allowing diffusion or active transport of maternal IgA to the child during pregnancy.

This notion is supported by previous work of Malek and colleagues [14], who investigated the transport of various proteins across human placenta by comparing the concentrations in maternal and fetal blood and where a correlation between fetal and maternal levels of serum IgA was found, suggesting that diffusion transfer does take place. Furthermore, Gurevich and colleagues [17] reported that endocrine precursor cells of 4–7 weeks old embryos were positive for surface IgA, but IgA-secreting lymphocytes did not appear until 10–11 weeks of pregnancy, suggesting a placental transfer of maternal IgA. Similarly, Ben-Hur and colleagues [28] found that components of the placental barrier were positive for IgA surface staining in 3.5–8 weeks old embryos, yet fetal, IgA-positive lymphocytes were first seen after 9–11 weeks of gestation. These findings collectively suggest that maternal IgA might be transferred to the embryo during pregnancy.

Some of the IgA-sufficient children born to IgAD mothers (group III) had detectable serum IgA levels at birth, implying that both the mother and the fetus contribute to the serum IgA detected in neonatal blood. Furthermore, the Am2 allotype has been shown to be present in the cord blood of some children, even though the mother lacks this marker, indicating a fetal origin of at least part of the neonatal serum IgA [12]. Based on our findings among the ten children in group III with detectable IgA levels, we suggest that the mother contributes at least 67% of the serum IgA detected in the DBSS eluates.

In summary, a screening approach for PID based on the determination of serum IgA levels in neonatal Guthrie card samples seems unlikely to be effective. However, it should be mentioned that the assay characteristics would be influenced by the prevalence of both the target diseases and those individuals with IgAD that give birth to healthy newborns; potentially proving the test to be of value following prospective evaluation. Alternatively, it remains to be determined if other markers, indicative of fetal IgA production, can be successfully applied to future neonatal screening programmes for PID.

## Materials and Methods

### Sample Collection

Guthrie card samples, generated within the first 72 hrs of life, were obtained from the PKU laboratory at the Centre for Inherited Metabolic Diseases, Karolinska University Hospital, Huddinge, Sweden. The samples were divided into three groups depending on origin. Group I: storage-time matched healthy individuals from the national neonatal biobank (n = 51). Group II: PID patients born to mothers with normal immunoglobulin levels (n = 31) or mothers with IgAD (n = 16). Group III: individuals with normal serum IgA levels born to IgA-deficient mothers (n = 20). Group II included 26 patients with IgAD, 11 newborns diagnosed with SCID (*RAG1* n = 3, *IL2RG* n = 3, *Jak3* n = 1, unknown etiology n = 4), 3 with HIGM (all CD40L mutations), 3 with XLA and 4 with A-T. The samples from these patients had all undergone TREC/KREC testing and were reported in our previous publications [26], [27]. Although all SCID, A-T and XLA patients could be readily identified, no IgAD or HIGM patients showed abnormal TREC or KREC copy numbers when compared to healthy newborns. The IgAD patients included in the present study had been referred to the immunodeficiency unit at Karolinska University Hospital Huddinge for evaluation of their immune status. As part of the investigation, all family members, including mothers, were also screened for humoral immunodeficiencies. All patients in group II lacked serum IgA at the time of investigation (n = 36) or at the time of hematopoietic stem cell transplantation (n = 11). Overall, the 118 DBSS in total had been stored for 2–28 years prior to analysis.

For this study, two spots (3.2 mm in diameter), each equaling the plasma fraction of ∼3 µl of whole blood, were punched from the original stored Guthrie cards. Approval for this study was obtained from the regional research ethical board in Stockholm.

### Comparison of different Elution Procedures

Peripheral venous blood was collected from four adult individuals and serum IgA levels determined by routine nephelometry. 15 µl of blood, corresponding to ∼9 µl of plasma, was spotted on twelve Whatman 903 protein saver cards (GE Healthcare, USA) per individual. The cards were left to dry and DBSS were subsequently eluted in 450 µl (1∶50) phosphate-buffered saline (PBS) with 0.5% Tween20 under varying conditions for temperature (4°C–37°C) and elution time (1 h –7 days). Serum IgA levels were determined by sandwich ELISA as described below. The ratio of IgA levels obtained after elution and the serum IgA levels determined by nephelometry was calculated and compared for each individual.

### Determination of IgA and IgG Levels in DBSS Eluates

The DBSS were soaked in 150 µl of PBS supplemented with 0.5% Tween20 (PBS-Tween, Sigma-Aldrich, USA) for 7 days at 4°C, giving a dilution of approximately 1∶50. 96-well polystyrene plates (Corning Incorporated, USA) were coated at room temperature overnight with 100 µl/well of polyclonal rabbit anti-human IgA or IgG antibodies (DAKO, Denmark) added at a dilution of 1∶4000. The DBSS eluates were diluted at 1∶5 and quantified using a serially diluted standard. Alkaline phosphatase-conjugated rabbit anti-human serum IgA or IgG antibodies (Jackson ImmunoResearch Laboratories, USA) were subsequently added at a 1∶2000 dilution, followed by the addition of a p-Nitrophenylphosphate solution (Sigma-Aldrich). The absorbance was measured at 405 nm on a Vmax microplate reader (Molecular Devices, USA) and the mean concentration was calculated for each sample using Deltasoft JV 1.8 (Biometallics Inc, USA). All included samples were tested 2–6 times.

### Statistical Analysis

The elution efficiency from DBSS was assigned by indicating a percentage agreement of those IgA levels measured by ELISA and the reference serum IgA levels measured by nephelometry. As the IgA levels in DBSS are not normally distributed, the data on the influence of storage time on IgA levels were analyzed according to Spearma?s rank correlation coefficient, using XLSTAT (Addinsoft, France). ROC curve analysis and calculation of test sensitivity, specificity, positive and negative predictive value was done using MedCalc (MedCalc Software, Belgium).
